# Characterization of the Activity Spectrum of MON 88702 and the Plant-Incorporated Protectant Cry51Aa2.834_16

**DOI:** 10.1371/journal.pone.0169409

**Published:** 2017-01-10

**Authors:** Pamela M. Bachman, Aqeel Ahmad, Jeffrey E. Ahrens, Waseem Akbar, James A. Baum, Scott Brown, Thomas L. Clark, Jennifer M. Fridley, Anilkumar Gowda, John T. Greenplate, Peter D. Jensen, Geoffrey M. Mueller, Matthew L. Odegaard, Jianguo Tan, Joshua P. Uffman, Steven L. Levine

**Affiliations:** 1 Monsanto Company, St. Louis, Missouri, United States of America; 2 Monsanto Company, Chesterfield, Missouri, United States of America; University of Tennessee, UNITED STATES

## Abstract

The spectrum of insecticidal activity of Cry51Aa2.834_16 protein targeting hemipteran and thysanopteran insect pests in cotton was characterized by selecting and screening multiple pest and non-pest species, based on representation of ecological functional groups, taxonomic relatedness (e.g. relationship to species where activity was observed), and availability for effective testing. Seven invertebrate orders, comprising 12 families and 17 representative species were screened for susceptibility to Cry51Aa2.834_16 protein and/or the ability of the protein to protect against feeding damage in laboratory, controlled environments (e.g. greenhouse/growth chamber), and/or field studies when present in cotton plants. The screening results presented for Cry51Aa2.834_16 demonstrate selective and limited activity within three insect orders. Other than *Orius insidiosus*, no activity was observed for Cry51Aa2.834_16 against several groups of arthropods that perform key ecological roles in some agricultural ecosystems (e.g. pollinators, decomposers, and natural enemies).

## Introduction

Hemipteran and thysanopteran pest species, including three species of plant bugs (the tarnished plant bug, *Lygus lineolaris Palisot de Bauvois*, the Western tarnished plant bug, *Lygus hesperus Knight*, and the cotton fleahopper, *Pseudatomoscelis seriatus* Reuter) and thrips, *Frankliniella* spp. are polyphagous pests of a wide range of agronomic and horticultural crops in various geographic regions [1–6]. With a high diversity of wild and cultivated hosts, populations of these pests increase throughout the growing season moving from host to host [7, 8]. Plant bugs and thrips were once considered secondary or minor sucking insect pests in cotton fields and have historically been controlled by numerous broad-spectrum insecticides delivered as foliar applications or seed treatments [6, 9]. However, changes in management of cotton pests and development of resistance to insecticides have resulted in these insects becoming major pests on transgenic *Bacillus thuringiensis* (*Bt*) cotton targeting key lepidopteran pests in the U.S., particularly in the mid-southern and southeastern growing areas [4, 10–12] and China [13].

Plant bugs and thrips negatively impact global cotton production, and currently present one of the biggest challenges for insect pest management in cotton [6, 14]. To prevent yield losses, the large populations of plant bugs and thrips found in cotton fields are now controlled with more frequent seed and foliar insecticide treatments at higher rates and with co-application of insecticides resulting in high costs to growers [6, 14, 15]. For the past few years, plant bugs have been ranked as the most important insect pests of cotton in the U.S. by the National Cotton Council and linked to the highest use of insecticide sprays for its control [16]. In the southeastern U.S. (e.g. Mississippi/surrounding states), control costs for *L*. *lineolaris* have approached or exceeded $100 per acre [17]. Likewise, thrips have been identified as the most significant “early season” pest of the U.S. cotton crop [6, 18] and the control options are limited with use of preventive seed treatment or foliar rescue sprays. The most common conventional chemical insecticides to control plant bugs and thrips are broad-spectrum insecticides such as pyrethroids, organophosphates, carbamates, and neonicotinoids [6, 16, 19].

Transgenic cotton expressing insecticidal *Bt* proteins have been globally adopted as a major tool for pest management in cotton since the first *Bt* cotton was first commercialized in 1996 in the U.S. [20, 21]. *Bt* cotton provides efficient control against three major lepidopteran cotton pests, tobacco budworms (*Heliothis virescens)*, bollworms (*Helicoverpa zea*), and pink bollworm (*Pectinophora gossypiella*). Currently available *Bt* cotton varieties are not effective against sucking pests because the *Bt* proteins present in the current products are only effective against lepidopteran pest species [22–24]. Recently, screening studies have identified *Bt* proteins showing significant dietary toxicity against hemipteran pests including *Lygus* plant bugs [25] and aphids [26, 27].

Monsanto Company has developed insect-protected cotton event MON 88702, which produces a modified *Bt* Cry51Aa2 insecticidal crystal (Cry) protein that protects against feeding damage caused by certain hemipteran and thysanopteran insect pests in cotton. This modified Cry51Aa2 protein, designated Cry51Aa2.834_16, has 96.44% sequence identity with the wild type *Bt* Cry51Aa2 [25], and shares structural similarities with the Cry35 protein expressed in DAS 59122 [28–30]. The modifications introduced into Cry51Aa2 resulting in Cry51Aa2.834_16 increased biological activity against hemipteran pests in the genus *Lygus* are described in detail in Gowda et al., [31]. Wide scale cultivation and use of commercialized biotechnology-derived crops expressing *Bt* Cry proteins, including Cry35, have revealed no instances of adverse impacts to non-target organisms (NTOs), and extensive reviews of the safety of various plant-incorporated Cry proteins are available [32, 33].

The mode of action (MoA) of the Cry51Aa2.834_16 protein has been characterized and includes the same general steps as other pore forming Cry proteins from *Bt*, including Cry1, Cry2, and Cry3, though the precise mechanisms are not known. [32, 34]. In brief, the biological activity and specificity of Cry51Aa2.834_16 is the result of the combined effects of achieving solubilization after ingestion of the protein by the target insect(s), proteolytic activation, binding to specific receptors on the insect midgut cell membrane, oligomerization, and pore formation [34, 35]. A more in depth discussion of the MoA of the Cry51Aa2.834_16 protein is provided in Jerga et al., [34]. Importantly, it is the combination of all of these steps (ingestion, activation and receptor-binding) that are required for activity, and thus result in a high degree of specificity [36].

The characterization of the activity spectrum of an insecticidal plant incorporated protectant (PIP) provides important information that informs the selection and scope of Tier 1 non-target organisms (NTOs) to be tested for an ecological risk assessment [37, 38]. The native Cry51Aa2 protein has been shown to share 97.7% sequence identity with the Cry51Aa1 protein described by Xu et al., [39] and both proteins are known to have activity against members of the Coleoptera [39]. Further, the native Cry51Aa2 protein was demonstrated to have activity at 500 ppm against two different hemipteran pest species, *L*. *lineolaris and L*. *hesperus*, and a coleopteran species, the Colorado potato beetle (*Leptinotarsa decemlineata*), but no activity against members of the order Lepidoptera [25].

To further elucidate the spectrum of activity of the Cry51Aa2.834_16 protein, an assessment of insecticidal activity was conducted against a range of surrogate invertebrates species in laboratory diet bioassays, controlled environments (e.g. greenhouse/growth chamber) using whole-plant bioassay, and field studies. Surrogate test species were selected based on representation for important ecological functional roles (e.g., detritivores, predators, parasitoids, pollinators), as well as phylogenetic relationship to pest species where activity had been observed.

Initially, the activity of the Cry51Aa2.834_16 against *L*. *hesperus* was evaluated in a 6-day laboratory bioassay. Additional greenhouse/growth chamber assays were performed with MON 88702 cotton plants expressing the Cry51Aa2.834_16 protein to evaluate biological activity against *L*. *lineolaris* as well as another hemipteran pest in the same family as *Lygus*, the cotton fleahopper, *P*. *seriatus* (Hemiptera; Miridae). As a part of the efficacy screening, field evaluations were conducted with MON 88702 cotton plants expressing Cry51Aa2.834_16 protein to evaluate activity against thrips which are members of the order Thysanoptera, a sister-group to Hemiptera, which together comprise the superorder Condylognatha [40].

To screen the biological activity of the Cry51Aa2.834_16 protein against a wider range of pest and non-pest species, continuous feeding laboratory studies were conducted against a series of invertebrates spanning six additional orders. A non-target beneficial predator, the insidious flower bug (*Orius insidiosus*: Hemiptera, Anthocoridae), from the same order but different family as the targets *Lygus* spp. and *P*. *seriatus*, was screened. Likewise, due to the observed activity of the native Cry51Aa2 protein to the coleopteran pest species *L*. *decemlineata* (Chrysomelinae; Chrysomelidae), five coleopteran species, including *L*. *decemlineata*, were screened with the Cry51Aa2.834_16 protein. These species were selected because they are closely related subfamilies and families to *L*. *decemlineata* and include representatives of the galerucines (Galerucinae; Chrysomelidae) *Diabrotica undecimpunctata howardi* and *Diabrotica virgifera virgifera*, and the coccinellids (Coccinellidae) *Epilachna varivestis* and *Coleomegilla maculata*.

Though no activity of the native Cry51Aa2 protein was previously observed against pest species in the order Lepidoptera, screening studies were conducted with the Cry51Aa2.834_16 protein against four additional lepidopteran species representing three families (*Ostrinia nubilalis*, Crambidae*; Plutella xylostella*, Plutellidae*;* and *Spodoptera frugiperda* and *Helicoverpa zea*, Noctuidae) to further confirm the absence of activity against insects in this order.

Additional screening bioassays were conducted against other ecologically relevant but more phylogenetically distant species including a representative pollinator and parasitoids, honey bees (*Apis mellifera*) and a parasitic wasp (*Pediobius foveolatus*), respectively from the order Hymenoptera, as well as representative decomposers, a soil hexapod (*Folsomia candida;* Collembola*)* and the earthworm *(Eisenia andrei;* Haplotaxida*)*.

## Materials and Methods

### Controlled environment/field studies using whole-plants

Controlled environment/field studies were conducted with transgenic cotton event MON 88702 and its non-transgenic near-isoline DP393. The control material has a genetic background similar to MON 88702 with the exception that it does not contain the inserted *Cry51Aa2*.*834_16* gene present in MON 88702. The field study was conducted under USDA notification for regulated trials with an academic cooperator on university-owned land (University of Mississippi, Sidon, MS).

#### *Lygus lineolaris* (Miridae: Hemiptera)

Cotton plants of MON 88702 and DP393 were established in 25 cm pots in a controlled environment growth chamber (16 h light at 32 ± 1°C and 8 h dark at 23 ± 1°C). At the peak squaring stage (~ 40 days after planting), plants were selected for use in the experiment that were approximately uniform in vigor, height, and growth stage. Individual plants were enclosed in a cage (98 cm x 140 cm) made from breathable plastic pollination sheets (Vilutis and Company Inc, Frankfort, IL) and then arranged in a completely randomized design with 5 replications (total 5 plants/treatment). Two pairs of sexually mature male and female *L*. *lineolaris* adults from laboratory culture (reared on organic beans) were released into each cage. Insects were allowed to mate, reproduce, and develop for 21 days. Thereafter, the numbers of next generation insects and their developmental stages were recorded on each plant. Nymphs younger or equal to 3^rd^ instar were recorded as small nymphs and 4^th^ and 5^th^ instars were recorded as large nymphs.

#### *Pseudatomoscelis seriatus* (Miridae: Hemiptera)

Cotton plants of MON 88702 and DP393 were established in 25 cm pots under greenhouse conditions (14 h light at 33 ± 2°C and 10 h dark at 27 ± 2°C). At the peak squaring stage (~ 40 days after planting), plants were selected and enclosed as stated above for *L*. *lineolaris* and then arranged in a completely randomized design with 12 replications (total 12 plants/treatment). Each caged plant was infested with three pairs of sexually mature *P*. *seriatus* and data were collected after four weeks of infestation and recorded as mentioned above for *L*. *lineolaris*.

#### *Frankliniella* spp. (Thripidae: Thysanoptera)

A field trial was conducted at Sidon, Mississippi to assess activity of cotton event MON 88702 against thrips and compared to DP393. The trial was planted on May 13, 2014 in a randomized complete block design with nine replications. Each plot consisted of eight rows 9.14 m long and a 76.2-cm row spacing, with ~4 seeds planted per foot. Plots were evaluated for thrips injury at 4–5 true leaf stage (June 20^th^, 2014). Damage to the cotton plants caused by thrips was assessed using a injury rating scale of 0–5 where 0 corresponded to no damage and no thrips observed; 1 corresponded to an indication of thrips being present but no visible injury symptoms; 2 corresponded to minor injury (slight crinkled and/or silvery areas) to the terminal bud and newly expanded leaves; 3 corresponded to a moderate injury (considerable crinkled and/or silvery areas) to the terminal bud and newly expanded leaves; 4 corresponded to severe injury (extensive crinkled and/or silvery areas) to the terminal bud and newly expanded leaves with some dead plants and aborted terminal buds; and 5 corresponded to plant death, severe stunting, stacked internodes, reduced leaf area and terminal bud abortion of most plants.

#### Data analysis

Effects of MON 88702 expressing Cry51Aa2.834_16 protein on numbers of next generation *L*. *lineolaris*, *P*. *seriatus* (sum of small nymphs, large nymphs, and adults) in caging trials, and thrips injury ratings in field trials were evaluated using PROC GLIMMIX and means were separated by the LSMEANS test (α = 0.05) using SAS^®^ [41].

### Laboratory diet bioassays

Direct feeding studies with both pest and non-pest insect species were used to evaluate the spectrum of insecticidal activity for the Cry51Aa2.834_16 protein. In total, fifteen key invertebrate species representing key ecological functional groups were evaluated in continuous feeding diet bioassays with the Cry51Aa2.834_16 protein. The test species covered 11 families and 6 orders, and included species from Hemiptera, Coleoptera, Lepidoptera, Hymenoptera, Collembola, and Haplotaxida. Direct feeding bioassays provide the ecologically relevant route of potential exposure to PIPs in the agronomic environment.

Laboratory bioassays were conducted with diet-incorporation methodology and insects were fed *ad libitum*. Where possible, bioassay procedures followed published guidelines or methods developed at the author’s laboratory and were initiated with the earliest life stages amenable to the assay design. Bioassays ranged from 6 to 28 days and evaluated lethal and sublethal endpoints to adequately characterize the potential for adverse effects. The endpoints evaluated included survival/mortality, and for some species included growth, development and/or reproduction. For species other than *Lygus* spp., the majority of Cry51Aa2.834_16 test concentrations were set at 200 and 400 μg/ ml or g diet. These concentrations were set based upon preliminary mean protein concentrations of Cry51Aa2.834_16 in field-grown cotton leaf tissues (~5 leaf stage) of approximately 200 μg/g fresh weight (data not shown) and therefore represent 1- and 2-fold of the potential field exposure levels based on leaf tissue. Cry51Aa2.834_16 concentrations greater than 400 μg/ml or g diet protein were selected for *D*. *v*. *virgifera* to evaluate the potential for effects at higher concentrations for this species.

All laboratory bioassays were conducted using a purified Cry51Aa2.834_16 protein produced in a recombinant *Bt* crystalliferous bacterium, and solubilized in 25 mM sodium carbonate/bicarbonate buffer (pH 10). A buffer control treatment that contained an equivalent level of carrier buffer in the test diet with the highest concentration of the Cry51Aa2.834_16 protein of the test material was included in each bioassay with the exception of *L*. *hesperus*. Where applicable, untreated assay controls (water only) and positive control treatments (e.g. potassium arsenate (KH_2_AsO_4_) were included to evaluate the performance of the assay and demonstrate the effectiveness of the dietary exposure system to detect toxic effects, respectively.

In total, four batches of purified Cry51Aa2.834_16 protein were used in the activity spectrum screening. All batches were confirmed using *L*. *hesperus* bioassays to be equipotent, and each batch of Cry51Aa2.834_16 used in the activity spectrum screening was confirmed for purity (100%) and identity (data not reported). Also, these four batches of *Bt*-produced Cry51Aa2.834_16 protein were evaluated to ensure that they were physicochemically equivalent based on sodium dodecyl sulfate polyacrylamide gel electrophoresis (SDS-PAGE) to estimate the purity and approximate molecular weight, and matrix-assisted laser desorption/ionization time-of-flight mass spectrometry (MALDI-TOF MS) to establish protein identity by peptide mapping.

#### Data analysis

Statistical analyses were conducted using either SAS [41] or GraphPad PRISM ^®^, Prism 6 for Windows [42] running under Windows 7. Clarification on the program used for each analysis is provided in the Supporting Information. PROBIT analysis with SAS [41] was used to estimated the dose level required for 50% mortality (LC_50_) for *L*. *hesperus*. For other species, survival and development were evaluated with Fisher’s Exact test (α = 0.05). The development days and biomass endpoints were statistically analyzed using an analysis of variance (ANOVA) (α = 0.05). For honey bee (*Apis mellifera*) larvae, the development time was calculated using SAS PROC MEANS program set at α = 0.05 [41]. SAS PROC MIXED was applied to fit model to conduct an ANOVA of the mean development time in each treatment. The pairwise comparisons between Cry51Aa2.834_16 and the assay control treatments and between Cry51Aa2.834_16 and the buffer control treatments were defined within the ANOVA and tested using *t*-test. For the springtail, *Folsomia candida*, the number of progeny were statistically compared using an analysis of variance (ANOVA) with a square root transformation (α = 0.05).

Endpoints are reported as means with standard errors.

#### *Lygus hesperus* (Miridae: Hemiptera)

*Lygus hesperus* were tested in 6-day continuous-feeding diet-incorporation bioassays to characterize the concentration-effect relationship and to estimate the LC50 value for the Cry51Aa2.834_16 protein. Three independent assays, each using a separate batch of insects, were performed. For each bioassay, the dose series was expected to elicit a response from *L*. *hesperus* nymphs that would allow for estimation of the LC_50_ value. *L*. *hesperus* eggs, oviposited in carrageenan-gel packs, were received from USDA Agricultural Research Service (ARS) Biological Control of Pests Research Unit (Stoneville, MS). For hatching, *L*. *hesperus* egg packs were placed in 64 oz plastic tub containers with a screened lid for ventilation and cultured in an incubator at 27°C, 60% relative humidity (RH), and a photoperiod of 14:10 h (L:D). During the hatching period of approximately 24 h, *L*. *hesperus* nymphs were provided an artificial *Lygus* diet (Bio-Serv, Frenchtown, NJ) mixed with whole chicken egg and water, encapsulated in Parafilm (Pechiney Plastic Packing, Chicago, IL) using a vacuum manifold system, herein referred to as ‘diet domes’. Each diet dome contained 150 μl of the *Lygus* diet. Diet domes were added to each hatch container during the acclimation period.

The biological activity of Cry51Aa2.834_16 protein on *L*. *hesperus* nymphs was evaluated with six treatment concentrations with a two-fold separation factor that ranged from 0.25 to 8.0 μg Cry51Aa2.834_16 protein/ml of diet. For incorporation of treatments into the *Lygus* diet, diet dosing solutions were first prepared by diluting the Cry51Aa2.834_16 protein solutions in purified water. The dosing solutions were then immediately incorporated at a ratio of 1:4 (w/v) with the base *Lygus* diet to prepare the treated diets.

Diet domes were prepared for each treated diet for separate dietary exposures to *L*. *hesperus* nymphs. Individual diet domes containing 150 μl of a test or control diet were placed into the wells of bioassay trays (128-well insect tray, #BAW128, Bio-Serv, Flemington, NJ). Using a vacuumed aspirator, the newly-hatched nymphs (≤ 26 h old) were transferred from a *Lygus* hatch container to the wells containing the diet dome treatments, targeting one insect per well and 32 insects per diet treatment to initiate the dietary exposure. The insects were contained in the bioassay wells with self-adhesive vented covers (#BACV16, Bio-Serv, Flemington, NJ); two additional vent holes were punched over each well using #2 insect pins (BioQuip, Rancho Dominguez, CA). The bioassays were incubated in an environmental chamber (Percival Scientific, Inc., Perry, IA) under the same conditions as described above for acclimation. After 6 days of continuous dietary exposure, *L*. *hesperus* nymphs were evaluated for survival/mortality and the percent survival was calculated for each test and control treatment group.

#### *Orius insidiosus* (Anthocoridae: Hemiptera)

*Orius insidiosus* nymphs were obtained from Koppert Biological Systems (Romulus, MI). Bioassay methods and insect handling procedures for *O*. *insidiosus* generally followed those as described in Tan et al., [43]. Five-day old *O*. *insidious* nymphs were exposed to the Cry51Aa2.834_16 protein incorporated into base diet at concentrations of 200 and 400 μg/g diet, respectively for a period of eleven days. The base diet consisted of 25% bee pollen, 25% *Ephestia kuehniella* eggs, and 50% water and was encapsulated in domes. A positive control treatment of potassium arsenate (KH_2_AsO_4_) at a concentration of 100 μg/g diet was also included. Each treatment consisted of 40 nymphs individually housed in petri dishes and fed *ad libitum* until all nymphs developed to adults or died. All nymphs hatched on the same day from the same batch of eggs were impartially assigned across treatments. Encapsulated diets were replaced every 48 hours and daily observations were made for survival and development to adulthood.

#### *Leptinotarsa decemlineata* and *Diabrotica* spp. (Chrysomelidae: Coleoptera)

*Leptinotarsa decemlineata* eggs were obtained from French Agricultural Research, Inc., (Lamberton, MN). First instar larvae (≤ 30 hours from hatching) of *L*. *decemlineata* were used to initiate dietary exposures to the test material Cry51Aa2.834_16 protein incorporated into an artificial agar-based CPB diet (BioServ, Frenchtown, NJ) at a concentration of 200 μg/ml of diet. Exposures to the control and test diets were initiated with a target number of 40 insects per diet treatment. Larvae were housed individually in 128-well bioassay trays (BAW128, BioServe, Frenchtown, NJ). Into each well, 0.5 ml of diet was dispensed which allowed the *L*. *decemlineata* larvae to feed *ad libitum*. Observations for survival were recorded for each insect on days 7 and 13. Throughout the duration of the exposure the *L*. *decemlineata* larvae were incubated in an environmental chamber at a target of 27°C temperature, 60% RH, with a photoperiod of 14:10h (L:D).

*Diabrotica spp*. eggs were obtained from Crop Characteristics, Inc. (Farmington, MN). The general methods for diet incorporation bioassay methods were used as described in Bolognesi et al., (2012). First instar larvae (≤ 30 hours from hatching) of *D*. *u*. *howardi* or *D*. *v*. *virgifera* were exposed to the Cry51Aa2.834_16 protein incorporated into an artificial agar-based diet at a concentration of 200 μg/ml (*D*. *u*. *howardi*) and 1000 μg/ml (*D*. *v*. *virgifera*) of diet. The treated diet mixture was dispensed in 0.25 ml aliquots into 32 wells (*D*. *u*. *howardi*) and 36 wells (*D*. *v*. *virgifera*) per treatment level in 48-well plates (Becton Dickson Labware) which allowed the larvae to feed *ad libitum*. Exposures to each of the test diets were initiated with a target number of 32 insects (*D*. *u*. *howardi*) and 36 insects (*D*. *v*. *virgifera*) per diet treatment. Observations for survival for each insect were recorded on day 7 (*D*. *v*. *virgifera*) and day 12 (*D*. *u*. *howardi* and *D*. *v*. *virgifera*).

#### *Coleomegilla maculata* (Coccinellidae: Coleoptera)

*Coleomegilla maculata* eggs were obtained from a culture held at the USDA Agricultural Research Service (ARS) (Beltsville, MD). First instar larvae (≤ 36 h from hatching) of *C*. *maculata* were used to initiate dietary exposures to Cry51Aa2.834_16 protein incorporated into an artificial agar-based pollen diet at two concentrations of 200 μg/ml and 400 μg/ml of diet, following the methods described in Bachman et al., [38]. A positive control was also included in which potassium arsenate (KH_2_AsO_4_) was incorporated at concentration of 100 μg/g diet. Exposures to each of the diets were initiated with a target total of 40 insects per diet treatment. The larvae were housed individually in petri dish test arenas and fed *ad libitum* with ~0.15 ml of respective test or control diet which administered every 48 h. Observations for mortality were made at each diet replacement until pupation (which ranged from 12–17 days from test initiation). The pupae were observed daily for adult emergence and adult *C*. *maculata* were weighed within 32 h of eclosion.

#### *Epilachna varivestis* (Coccinellidae: Coleoptera)

*Epilachna varivestis* eggs were obtained from the New Jersey Department of Agriculture, Philip Alampi Beneficial Insect Rearing Facility (Trenton, NJ). First instar larvae (< 24 h after the first observation of hatching) of *E*. *varivestis* were used to initiate dietary exposures to an agar-based modified SCR diet (Bio-Serv, Frenchtown, NJ) containing Cry51Aa2.834_16 protein at 200 μg/ml and 400 μg/ml of diet for a period of 28 days following the methods described in Bachman et al., [38]. Two positive control diets were also included, in which potassium arsenate (KH_2_AsO_4_) was incorporated at the nominal concentrations of 14 μg/g diet and 28 μg/g diet. Exposures to each of the diets were initiated with a target total of 40 insects per diet treatment and larvae were housed individually in 128-well bioassay trays (BAW128, BioServe, Frenchtown, NJ). Into each well 0.25 ml of diet was dispensed which allowed the *E*. *varivestis* larvae to feed *ad libitum*. Fresh diet was provided to each surviving larva every 7 days by careful transfer of larvae to a new well of a bioassay tray containing the respective diet treatment. Observations for survival were recorded for each insect on test days 7 and 14.

#### *Ostrinia nubilalis* (Crambidae: Lepidoptera); *Helicoverpa zea* and *Spodoptera frugiperda* (Noctuidae: Lepidoptera); and *Plutella xylostella* (Plutellidae: Lepidoptera)

*Ostrinia nubilalis* eggs were obtained from an in-house culture (Waterman, IL). Eggs from *H*. *zea*, *S*. *frugiperda*, and *P*. *xylostella* were obtained from Benzon Research Inc. (Carlisle, PA). All bioassays with lepidopteran species were initiated with first instar larvae ≤ 30 h from hatching. Larvae of *H*. *zea*, *S*. *frugiperda*, *O*. *nubilalis*, and *P*. *xylostella* were exposed to the test material Cry51Aa2.834_16 protein incorporated into the artificial agar-based diet (Southland multi-species diet, Lake Village, AR) at a concentration of 400 μg/ml of diet. For *H*. *zea*, *S*. *frugiperda*, and *O*. *nubilalis* each treatment diet was initiated with a target number of 16 insects per diet treatment and the Cry51Aa2.834_16 protein treated diets were replicated 2 times for a target total of 32 larvae for each of the test diets. For *P*. *xylostella*, each treatment diet was initiated with a target number of 32 insects per diet treatment and the Cry51Aa2.834_16 protein treated diets were replicated 2 times for a target total of 64 larvae for each of the test diets. The larvae were housed individually in 128-well bioassay trays (BAW128, BioServe, Frenchtown, NJ). Into each well 1.0 ml (*H*. *zea*, *S*. *frugiperda*, and *P*. *xylostella)* or 0.5 ml (*O*. *nubilalis*) of diet was dispensed which allowed the larvae to feed *ad libitum*. Throughout the exposure duration the all lepidopteran larvae were incubated in an environmental chamber at a target temperature of 27°C, a target RH of 60%, and a photoperiod of 14:10h (L:D). Observations for survival were recorded for each insect per treatment on day 7.

#### *Pediobius foveolatus* (Eulophidae: Hymenoptera)

*Pediobius foveolatus* pupae (in parasitized *E*. *varivestis* larvae) were obtained from the New Jersey Department of Agriculture, Philip Alampi Beneficial Insect Rearing Facility (Trenton, NJ). Bioassay methods and insect handling procedures for *P*. *foveolatus* generally followed those as described in Bachman et al., [38]. One-day old adult wasps were exposed to Cry51Aa2.834_16 protein incorporated into 30% honey/water (v/v) solution at concentrations of 200 μg/ml and 400 μg/ml, respectively, for a period of 20 days. Potassium arsenate (KH_2_AsO_4_) at a concentration of 200 μg/ml diet was used as the positive control substance. All controls consisted of three replicates and Cry51Aa2.834_16 treatments included 2 replicates. Each replicate included at least 20 individual adults housed in a flask (162 cm^2^ cell culture flask with vented cap, Costar^®^ 3151, Corning Inc., Corning, NY) and provided with appropriate treatment diet in two screened feeding dishes. The test and control diets were replaced every 48 h at which time mortality was recorded.

#### *Apis mellifera* (Apidae: Hymenoptera)

Larvae of *A*. *mellifera*, approximately 2 days old (California Agricultural Research, Inc, Kerman, CA), were exposed to Cry51Aa2.834_16 protein in a single dose administered to the brood cell following methods in Tan et al., [44]. A single dose of 2 mg Cry51Aa2.834_16 protein/ml diet solution in 10 μL aliquot of 30% (w/v) sucrose/purified water was added to each larval cell at test initiation for a total mass of 20 μg Cry51Aa2.834_16/larvae. Each treatment consisted of two replicates and each replicate tested 20 honey bee larvae. For each replicate, two frames from a single hive were selected for the Cry51Aa2.834_16 protein, assay control (untreated), positive control (KH_2_AsO_4_ at 2 mg/ml) treatments. One frame from a separate hive was used for the buffer control treatment. Each side of the 2 frames from the hives was impartially assigned to each of the treatments. Upon completion of diet solution administration, frames were held in the insulated container for at least 30 minutes before being returned to their original hive.

Observations were made at 6 days after dosing by removing the treated frames from their respective hives and evaluating for capping. Once the evaluation was completed the frames were returned to their respective hives. On day 12 after dosing, the frames were once again removed from their hives, capping was rechecked and the frames placed in a growth chamber. All frames were then moved into a screened hive box and placed in a growth chamber under a photoperiod of 0:24h (L:D). Temperature and RH ranged from 23.8 to 30.7°C and 50.7% to 63.3%, respectively. On days 13 through 17 after dosing, daily evaluations were conducted for adult emergence; this observation interval encompassed the adult emergence of all surviving larvae. A statistical analysis was not performed since there was 100% survival in the Cry51Aa2.834_16 treatment, assay control, and buffer control.

#### *Folsomia candida* (Isotomidae: Collembola)

In a study based upon OECD Guideline 232 [45], survival and reproduction of the springtail (*F*. *candida*) was evaluated via dietary exposure to an inactivated yeast diet. The test arenas consisted of lidded glass jars, lined at the base with a solid layer of a plaster-of-Paris and charcoal substrate. Groups of 10 juvenile *F*. *candida* (9–10 days old; Mambo-Tox Ltd, Southampton, UK) were placed into each replicate jar. Four replicates of 10 *F*. *candida* were included for each diet treatment, and diets were renewed every 2–3 days. Throughout the study, the test arenas were maintained in a controlled-environment cabinet from 19.6–20.1°C, 65–71% RH, with a photoperiod of 16:8h (L:D) and lighting of 450–650 lux. At 28 days, the numbers of the original population of *F*. *candida* surviving in each test arena and the numbers of their offspring were recorded for each replicate arena. The numbers of surviving adults were used to calculate the percentage mortality of the *F*. *candida* originally introduced in each treatment.

#### *Eisenia andrei* (Lumbricidae: Haplotaxida)

*Eisenia andrei* (Mambo-Tox Ltd, Southampton, UK) were exposed to Cry51Aa2.834_16 protein incorporated into a standard artificial soil medium at 400 μg Cry51Aa2.834_16/g soil dry weight for 14 days in a study based upon OECD Guideline 207 [46]. Control and Cry51Aa2.834_16 treatments were incorporated into an artificial soil substrate containing 10% w/w peat, the moisture content of which was brought to 50% of its pre-determined maximum water-holding capacity by the addition of the individual treatments. The treated soil was placed into 1-L lidded jars. The study included 4 replicates per treatment with 10 adult *E*. *andrei* (approx. 5 months old, with a fresh weight of 300–600 mg and with a visible clitellum) introduced into each jar. No additional food was provided during the duration of the test. During the bioassy, all jars were maintained at 19.4–20.1°C, a photoperiod of 24:0h (L:D), and illumination of 490–620 lux. Survival was assessed over a 14-day test period and the change in fresh weight of the worms was assessed for survivors at 14 days after treatment. Percent change in weight of the worms for the two controls and Cry51Aa2.834_16 treatment over the 14-day bioassay was calculated. The overall percent change in mean-weight per replicate was derived by comparing the mean start- and end-weights for worms in each replicate.

## Results

### Controlled environment/field studies using whole-plants

#### Lygus lineolaris

**Fig 1:**
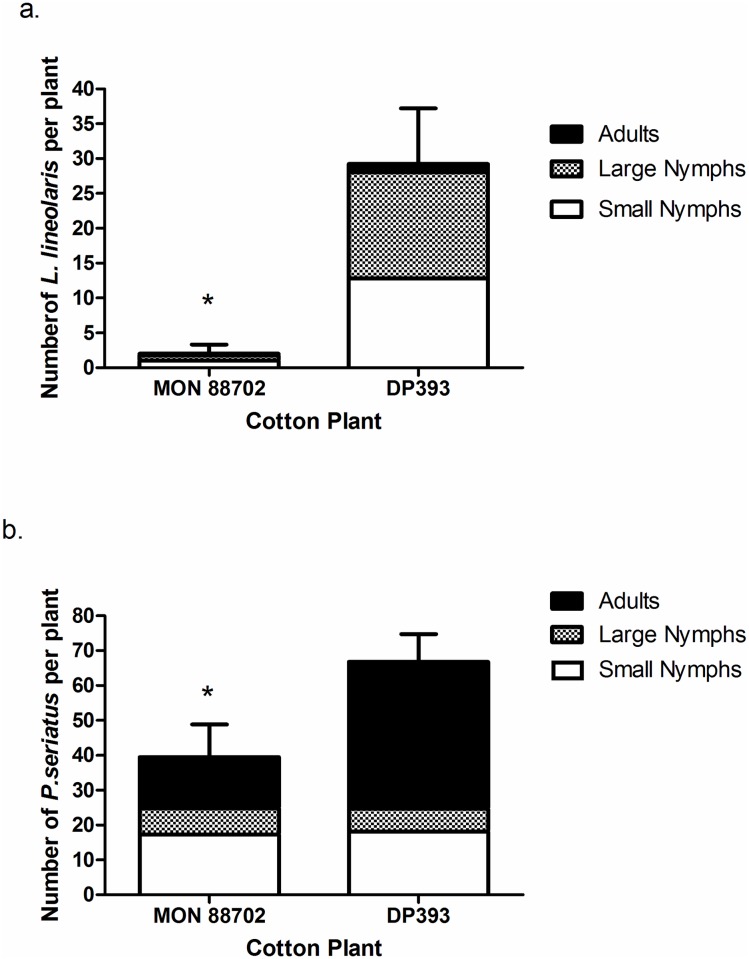
Numbers and stage of next generation (a) *L*. *lineolaris* and (b) *P*. *seriatus* recovered from MON 88702 cotton expressing Cry51Aa2.834_16 protein in whole plant caging experiments. Standard error bars are for total numbers of next generation insects. *indicates a significant difference (α = 0.05) between MON 88702 and DP393 (non-transgenic near-isoline).

A high level of efficacy was recorded for MON 88702 when tested against L. lineolaris. The majority of next generation L. lineolaris on MON 88702 were small nymphs (3^rd^ instar or younger) whereas on DP393 plants more large nymphs (4^th^ and 5^th^ instars) were observed (Fig 1a). There was a 19-fold reduction in numbers of large nymphs, the economically most important stage, recovered from MON 88702 compared to DP393 (F = 16.35; df = 1, 4; p = 0.016). Differences were not detected in small nymphs (F = 3.92; df = 1, 4; p = 0.119) and adults (F = 1.5; df = 1, 4; p = 0.29) between MON 88702 and DP393; however, the comparison of total number of next generation of L. lineolaris (sum of all developmental stages) also revealed 15-fold reduction on MON 88702 plants (F = 11.25; df = 1, 4; p = 0.028).

#### Pseudatomoscelis seriatus

On MON 88702 plants the majority of *P*. *seriatus* were small nymphs, while on DP393 plants the majority of *P*. *seriatus* developed to adult stage (Fig 1b). While no significant differences were found between the numbers of small and large nymphs on MON 88702 and DP393, there was a significant 3-fold reduction in numbers of next generation adults recorded between MON 88702 and DP393 (*F* = 53.81; df = 1,9; p *<* 0.001). This resulted in a 1.7-fold reduction in total number of next generation *P*. *seriatus* on MON 88702 plants (*F* = 16.86; df = 1, 9; p *=* 0.003).

#### *Frankliniella* spp.

**Fig 2:**
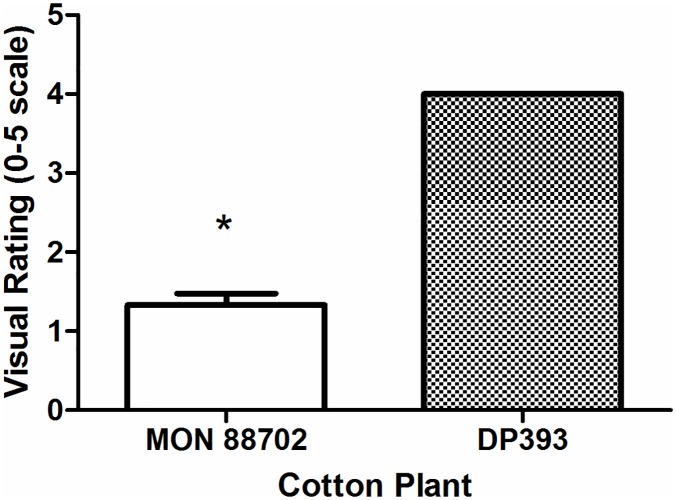
Thrips injury rating of MON 88702 expressing Cry51Aa2.834_16 protein under field conditions. *indicates a significant difference (α = 0.05) between MON 88702 and DP393 (non-transgenic near-isoline).

High thrips pressure was recorded in the field trial as all plots of DP393 plants showed severe injury to the terminal bud and leaves with some dead plants and aborted terminal buds whilst MON 88702 plants showed minimal injury (Fig 2). Under these high pressure conditions, MON 88702 plants showed only minimal thrips presence and significantly less injury to terminal buds and leaves (*F* = 341; df = 1, 8; p *<* 0.001).

### Laboratory diet bioassays

A summary of the results from the laboratory diet bioassays, controlled environment studies, and field studies is provided in Table 1 and additional information for each laboratory study is supplied in Supporting Information S1 File.

**Table 1:** Activity Screening results from feeding assays with Cry51Aa2.834_16 protein in invertebrates representing target and non-target species.

Order	Family	Species	Representative Function	Bioassay Duration (days)	Endpoints	Mean LC_50_ or Maximum Concentration (μg/ml or g)(mg(mg/Tested^2^(mg/mL or g diet)	Activity
Hemiptera	Miridae	*Lygus hesperus*	Herbivore	6	S	3.0	Yes
		*Lygus lineolaris*	Herbivore	21	S, D	Plant expression	Yes
		*Pseudatomoscelis seriatus*	Herbivore	28	S, D	Plant expression	Yes
	Anthocoridae	*Orius insidiosus*	Predator	11	S, D	400	Yes^1^
Thysanoptera	Thripidae	*Frankliniella* spp.	Herbivore	37	Severity of damage	Plant expression	Yes
Coleoptera	Chrysomelidae	*Leptinotarsa decemlineata*	Herbivore	13	S	200	Yes^2^
	Chrysomelidae	*Diabrotica undecimpunctata howardi*	Herbivore	12	S	200	Yes^3^
		*Diabrotica virgifera virgifera*	Herbivore	12	S	1000	No
	Coccinellidae	*Coleomegilla maculata*	Predator	17	S, G, D	400	No
	Coccinellidae	*Epilachna varivestis*	Herbivore	28	S	400	No
Lepidoptera	Noctuidae	*Helicoverpa zea*	Herbivore	7	S	400	No
	Noctuidae	*Spodoptera frugiperda*	Herbivore	7	S	400	No
	Crambidae	*Ostrinia nubilalis*	Herbivore	7	S	400	No
	Plutellidae	*Plutella xylostella*	Herbivore	7	S	400	No
Hymenoptera	Eulophidae	*Pediobius foveolatus*	Parasitoid	20	S	400	No
	Apidae	*Apis mellifera* (larvae)	Pollinator	17	S, D	20 μg/larvae	No
Collembola	Isotomidae	*Folsomia candida*	Decomposer	28	S, R	400	No
Haplotaxida	Lumbricidae	*Eisenia andrei*	Decomposer	14	S, G	400	No

S = Survival, G = Growth, D = Development, R = Reproduction

^1^ The survival response was 66.7% in the 400μg mCry51Aa2.834_16/ml diet treatment which was the highest concentration tested for *O*. *insidiosus*.

^2^The corrected survival response was 50.0% in the 200μg mCry51Aa2.834_16/ml diet treatment for *L*. *decemlineata*.

^3^The corrected survival response was 54.8% in the 200μg mCry51Aa2.834_16/ml diet treatment for *D*. *u*. *howardi*.

#### Lygus hesperus

The mean six-day LC_50_ value for *L*. *hesperus* exposed to diet incorporated with Cry51Aa2.834_16 protein was determined to be 3.0 ± 0.4 μg Cry51Aa2.834_16 /ml diet (Table A in S1 File). The PROBIT model fit the data well and the dose -responses for each bioassay were monotonic (Fig A in S1 File).

#### Orius insidiosus

The survival of *O*. *insidiosus* nymphs fed the buffer control diet was 98% and all surviving nymphs developed to normal adults, indicating a negligible background effect. The total survival on the Cry51Aa2.834_16 treatments was significantly different (67%, p = 0.001) for both concentrations of 200 and 400 μg/g diet (Table B in S1 File). However, the developmental times between Cry51Aa2.834_16 diet treatments and the buffer control were not different (Fig B in S1 File). The mean development time on the 200 μg Cry51Aa2.834_16 /g diet treatment and 400 Cry51Aa2.834_16 μg/g diet treatments were not significantly different from the buffer control and were estimated to be 10.9 ± 0.2 d (p = 0.149), and 10.9 ± 0.2 d (p = 0.141), respectively. Likewise, the mean development time on the buffer control treatment was estimated to be 10.5 ± 0.1 d. In the positive control treatment of 100 μg KH_2_AsO4/g diet the effectiveness of the test system to detect toxic effects was confirmed, as nymph mortality began at day 3, reached 95% by day 7, and no surviving adults were observed in this treatment. (Table B in S1 File). These results demonstrate that continuous chronic dietary exposure to Cry51Aa2.834_16 protein for 11 days, at 200 and 400 μg/g diet, impacted survival of *O*. *insidiosus* nymphs. However, the development and development time of surviving nymphs to adults was not adversely affected at both Cry51Aa2.834_16 test concentrations.

#### *Leptinotarsa decemlineata* and *Diabrotica* spp.

The survival of *L*. *decemlineata* larvae fed the assay control (92%) and buffer control (92%) were not significantly different (p = 1.00) indicating a negligible background effect. On the 200 μg Cry51Aa2.834_16/ml treatment, *L*. *decemlineata* survival (50%) was significantly different from the combined control (p < 0.001) (Table C in S1 File). The results for the 7-day continuous dietary exposure to Cry51Aa2.834_16 protein at a concentration of 200 μg/ml, demonstrate an adverse effect on the survival of *L*. *decemlineata* larvae.

The survival of *D*. *u*. *howardi* larvae on the buffer control (85%) was significantly different from the 200 μg Cry51Aa2.834_16/ml treatment (55%; p = 0.001) (Table C in S1 File). The results for the 12-day continuous dietary exposure to Cry51Aa2.834_16 protein at a concentration of 200 μg/ml demonstrate an adverse effect on the survival of *D*. *u*. *howardi* larvae.

The survival of *D*. *v*. *virgifera* larvae at day 7 (97%) and day 12 (74%) on the buffer control indicated an acceptable background effect. *D*. *v*. *virgifera* survival on the 1000 μg/ml Cry51Aa2.834_16 treatment was not significantly different from survival on the buffer control on both day 7 (100%; p = 0.571) and day 12 (78%; p = 0.811) (Table C in S1 File). The results for the 12-day continuous dietary exposure to the Cry51Aa2.834_16 protein, at the concentration of 1000 μg/ml, demonstrate no observed adverse effects on the survival and growth of *D*. *virgifera virgifera* larvae.

#### Coleomegilla maculata

The *C*. *maculata* survival in the assay control (78%) and buffer control (82%) was not significantly different (p = 0.781). *C*. *maculata* survival in both Cry51Aa2.834_16 treatments at 200 μg /ml (88%, p = 0.546) and 400 μg/ml (93%, p = 0.193) was not significantly different from the control treatments indicating no adverse effects of Cry51Aa2.834_16 on survival (Table D in S1 File). The percent adult emergence was not significantly different (p = 0.586) between the assay control (78%) and buffer control (82%), nor between the buffer control and Cry51Aa2.834_16 treatments at 200 μg /ml (88%, p = 0.546) and 400 μg /ml (85%, p = 0.770).

Consistent with the survival results, the mean number of days to adult emergence was not significantly different (p = 0.623) between the assay control (16.2 ± 1.3 days), buffer control (15.8 ± 1.1 days), and Cry51Aa2.834_16 treatments at 200 μg /ml (15.9 ± 1.1 days) and 400 μg/ml (16.1 ± 1.0 days) (Fig C in S1 File). Mean adult mass was also not significantly different (p = 0.508) between the assay control (9.2 ± 1.6 mg), buffer control (9.7 ± 1.4 mg), and Cry51Aa2.834_16 treatments at 200 μg /ml (9.7 ± 1.0 mg) and 400 μg/ml (9.6 ± 1.6 mg). (Table D in S1 File). The survival on the positive control was 35% and none of the larvae on this treatment developed to the pupal stage, confirming the effectiveness of the test system to detect toxic effects (Table D in S1 File). These results demonstrate no observed adverse effects on the survival growth and development of *C*. *maculata* larvae through adult eclosion with continuous chronic dietary exposure to Cry51Aa2.834_16 protein at both tested concentrations.

#### Epilachna varivestis

The *E*. *varivestis* survival at day 7 and day 14 on the assay control (100%, 90%) and buffer control (98%, 93%) were not significantly different on day 7 (p = 0.494) or day 14 (p = 1.00) indicating negligible background mortality. *E*. *varivestis* larval survival was not significantly different between the buffer control on day 7 (98%) and the 200 μg/ml (100%, p = 1.00) or 400 μg/ml (100%, p = 1.00) Cry51Aa2.834_16 treatments. *E*. *varivestis* larval survival was also not significantly different between the buffer control on day 14 (93%) and the 200 μg/ml (98%, p = 0.615) or 400 μg/ml (95%, p = 1.00) Cry51Aa2.834_16 treatments. (Table E in S1 File). In contrast, survival on the positive control treatment (3%) day 14 was lower confirming the effectiveness of the test system to detect toxic effects. These results demonstrate no observed adverse effects on the survival of *E*. *varivestis* larvae with continuous chronic dietary exposure over 14 days to Cry51Aa2.834_16 protein at concentrations tested up to 400 μg /ml of diet.

#### *O*. *nubilalis*, *H*. *zea*, *S*. *frugiperda*, and *P*. *xylostella*

For all lepidopteran species tested, no significant differences were found between the assay and buffer control survival (p = 1.00) in the individual studies. Therefore, the controls were combined within individual studies for comparison against the 400 μg Cry51Aa2.834_16/ml diet treatments for each species (Table F in S1 File). The survival of *O*. *nubilalis* larvae was not significantly different (p = 0.484) on the Cry51Aa2.834_16 treatment (97%) when compared to the 100% survival in combined controls at day 7. Likewise, the day 7 survival of *H*. *zea* (97%), *S*. *frugiperda* (100%), and *P*. *xylostella* (100%) were not significantly different (p = 1.00 for each study) when compared to the combined. These results demonstrate no observed adverse effects on the survival after of these lepidopteran species after 7-days of continuous dietary exposure to Cry51Aa2.834_16 protein a concentration of 400 μg/ml of diet.

#### Pediobius foveolatus

The survival of *P*. *foveolatus* wasps on the assay control (100%) and buffer control (97%) was not significantly different (p = 0.238), indicating no background effect on survival of test adults. Survival on the Cry51Aa2.834_16 treatments was not significantly different from the buffer control at 200 μg/ml (100%, p = 0.509) and 400 μg/ml (94%, p = 0.649) (Table G in S1 File). In contrast, on the positive control treatment of 200 μg KH_2_AsO_4_/ml diet, mortality was observed at day 4 and reached 100% by day 12 confirming the effectiveness of the test system to detect toxic effects. These results demonstrate no observed adverse effects on the survival after 20 days to *P*. *foveolatus* adults with 20-days of continuous dietary exposure to Cry51Aa2.834_16 protein at concentrations tested up to 400 μg/ml of diet.

#### Apis mellifera

The survival of *A*. *mellifera* larvae was 100% on the Cry51Aa2.834_16 treatment at a concentration of 2000 μg /ml diet solution, as well as on the assay control (30% sucrose) and buffer control treatments. The single dose of 10 μl of 2000 μg /ml solution added to each larval cell equates to a total mass of 20 μg Cry51Aa2_16/larvae. The positive control treatment of potassium arsenate at 2000 μg /ml (20 μg/larvae) had 0% survival, confirming the effectiveness of the test system to detect toxic effects (Table H in S1 File). The mean development time on the Cry51Aa2.834_16 treatment (14.3 ± 0.2 days) was not significantly different from the assay control (14.3 ± 0.2 days; p = 0.969) or the buffer control (14.7 ± 0.8 days; p = 0.496) (Fig D in S1 File). Most importantly, the Cry51Aa2.834_16 treated bees initiated and completed emergence in sync with the control bees. These results demonstrate no observed adverse effects on the survival and development time of *A*. *mellifera* larvae after dietary exposure to Cry51Aa2.834_16 protein at 20 μg Cry51Aa2.834_16/larvae.

#### Folsomia candida

At 28 days, the percent survival for adult *F*. *candida* on the assay and buffer controls diets were both 100% and therefore these treatments were combined for statistical comparisons with the treatment. There were no significant differences in survival (p = 0.333) between the combined control and the 400 μg Cry51Aa2.834_16/g diet treatment (97%) (Table I in S1 File). The blank (starvation) control resulted in 100% survival; however, *F*. *candida* were very small compared to the buffer and assay control diet treatment *F*. *candida*. In the toxic reference diet treatment, 17% survival was recorded confirming the effectiveness of the test system to detect toxic effects.

The mean number of progeny produced per replicate was not significantly different (p = 0.505) between the assay control diet (170 ± 2), the buffer control diet (161± 2) and therefore these controls were combined. Likewise, there was no significant difference (p = 0.553) between the Cry51Aa2.834_16 protein diet treatment (159 ± 7) and the combined controls (165 ± 3). (Table I in S1 File). On the blank (starvation) control, a mean of 2 progeny were produced per replicate. This effect of the blank (starvation) control on reproduction, demonstrates that the yeast diet was necessary for the *F*. *candida* to grow and reproduce under these test conditions. Also, it was noted that the original *F*. *candida* added to the blank control arenas at the start of the test remained small compared to the untreated control. On the toxic-reference diet treatment, the mean number of progeny produced per replicate was 4, indicating this assay was effective for detecting toxic effects through dietary exposure.

These results indicate that 400 μg Cry51Aa2.834_16 protein/g inactive-yeast diet had no observed adverse effects on the survival or reproductive capacity of *F*. *candida*.

#### Eisenia andrei

At 7 and 14 days, worms in all the treatments appeared healthy and active. After 14 days, survival of the *E*. *andrei* was 100% in all treatments (Table J in S1 File). The mean percentage change in *E*. *andrei* worm weight was not significantly different (p = 0.743) between the assay control (9.4%), the buffer control (6.7%) and therefore these controls were combined for comparison with the test substance. There was no significant difference (p = 0.113) between earthworm weight in the 400 μg Cry51Aa2.834_16/g soil treatment (6.2%) and the combined controls. These results indicate no observed adverse effect of 400 μg Cry51Aa2.834_16/g soil dry weight on earthworm survival or body weight after 14 days of continuous exposure.

## Discussion

Seventeen invertebrate species representing key ecological functional roles most relevant for agricultural systems [37, 47] were screened for susceptibility to Cry51Aa2.834_16 protein and/or the ability of the protein to protect MON 88702 plants against feeding damage in laboratory, controlled environments (e.g. greenhouse/growth chamber), and/or field evaluations when expressed in cotton plants. The range of surrogate invertebrates screened was selected based on multiple factors, including ecological and economic importance, representation of different habitats (below ground, ground dwelling, above ground), representation of valued taxa or functional groups (e.g. predator, parasitoids, decomposers, or pollinators), taxonomic relatedness (e.g. relationship to species where activity was observed), and availability for effective testing in the laboratory. This screening approach is routinely used in ecological risk assessment (ERA), and was used for evaluation of several previously approved and commercialized PIPs [37, 47–50]. These results provide an initial assessment of Cry51Aa2.834_16 against both target pests and NTOs, thereby providing insight into future tests needed to evaluate potential for unintended adverse effects under field conditions.

Insecticidal activity of Cry51Aa2.834_16 was detected against several pest species from three different orders, including Hemiptera, Thysanoptera, and Coleoptera. The hemipteran target species *L*. *hesperus* in the family Miridae demonstrated sensitivity in diet incorporation bioassays with a mean LC_50_ of 3.0 μg/ml diet. Although this LC_50_ value is higher than median effect concentrations reported for highly susceptible lepidopteran species to Cry1 and Cry2 proteins it is comparable with reported median effect concentrations for other commercialized *Bt* proteins used for control of rootworms [51–53]. Likewise, greenhouse and growth chamber studies confirmed the activity of the Cry51Aa2.834_16 protein expressed in cotton plants against two other hemipteran mirid species, *L*. *lineolaris* and *P*. *seriatus*. The results of these studies with *L*. *hesperus* and *L*. *lineolaris* are consistent with the results reported in Gowda et al., [31] where lethal effects of the Cry51Aa2.834_16 protein were reported for each of these species respectively.

A third hemipteran representative, from the family Anthocoridae, *O*. *insidiosus*, presented adverse effects from the Cry51Aa2.834_16 protein at 200 μg/ml diet. The presence of activity within *O*. *insidiosus* is not unexpected in a direct feeding bioassay with artificial diet, as the species is closely related to the hemipteran target pests. As for other predators in the cotton agroecosystem, *O*. *insidiosus* is expected to have limited exposure to the Cry51Aa2.834_16 protein through the consumption of prey, as previous studies have shown limited biotransfer of Cry proteins between trophic levels [54, 55] due to the degradation and/or dilution of the Cry proteins in the gut of species without the multiple conditions and receptors necessary for toxicity. Therefore, due to their feeding ecology, this species is not expected to encounter levels of the Cry51Aa2.834_16 protein in the field that will likely result in an adverse biological effect.

Additionally, field evaluation results using plants expressing Cry51Aa2.834_16 demonstrated protection against feeding damage from thrips (*Frankliniella* spp.), in the order Thysanoptera. Given that the order Hemiptera and Thysanoptera are closely related and together comprise the superorder Condylognatha, protection against feeding damage from thrips is also not unexpected [56].

Within Coleoptera, the Cry51Aa2.834_16 protein showed a similar level of effect on survival of *L*. *decemlineata* and *D*. *u*. *howardi* larvae at 200 μg/ml diet. This is expected, as native Cry51Aa1 and native Cry51Aa2 (TIC807), both of which have greater than 95% sequence identity with Cry51Aa2.834_16, also showed activity against *L*. *decemlineata* [25, 39]. However, no toxicity was observed toward larvae of a closely related species *D*. *v*. *virgifera* within the same family (Chrysomelidae). Differential activity between *D*. *u*. *howardi* and *D*. *v*. *virgifera* has also previously been observed with the corn rootworm active eCry3.1Ab protein [51]. Additionally, two other coleopteran species from the more basal family Coccinellidae, *C*. *maculata* and *E*. *varivestis*, showed no sensitivity to the Cry51Aa2.834_16 protein at similar concentrations. These screening results indicate that although Cry51Aa2.834_16 shows activity against certain members of the insect order Coleoptera, activity may be restricted, to some members of the Chrysomelidae family.

Additional screening studies with representatives of the orders Lepidoptera, Hymenoptera, Collembola, and Haplotaxida demonstrated no observed adverse effects from continuous dietary exposure to the Cry51Aa2.834_16 protein, indicating that the activity of the Cry51Aa2.834_16 protein toward invertebrate species is selective, and therefore limited. The taxa from these orders are especially important as they serve as key NTOs utilized for the ERA of PIPs [47, 57], and confirm that the activity of Cry51Aa2.834_16 is selective and the likelihood of adverse effects to NTOs is low.

A key feature that makes Cry proteins attractive for pest management in crops is their ability to provide protection against insect feeding damage, but with a high degree of selective activity [36]. In general, Cry proteins have a narrow spectrum of insecticidal activity within a particular insect order [22], although there are an increasing number of Cry proteins that have been observed to show cross-order activity [58]. As examples, Cry2Aa (formerly CryB1) is active against insects from the orders Lepidoptera and Diptera (mosquitoes) [22, 23, 58], while Cry3Aa displays activity against insects from the orders Coleoptera, Hemiptera, and Hymenoptera [58]. It is important to know that this cross-order activity does not impact the environmental safety of *Bt*-based products as Cry proteins tend be highly toxic to one order or closely related orders (primary specificity range), but much less toxic to distantly related taxa [36, 58, 59]. This pattern of activity was observed for the Cry51Aa2.834_16 protein where high toxicity or protection from feeding damage was shown against some hemipterans and thysanopteran species (primary specificity range), but relatively weak toxicity was shown against distantly related Coleoptera species.

These observations do not imply that Cry proteins with cross-order activity lack selectivity; rather, the necessary conditions to enable activity of these Cry proteins, including proteolytic activation and target receptor binding, are likely present in susceptible species across insect orders. The specificity of the protein—and therefore selective activity against insects—of these *Bt* proteins is believed to derive from a combination of their relative solubility, stability, proteolytic activation, and receptor binding on the insect midgut epithelium [35]. Jerga et al., [34] recently confirmed that the mode of action of the Cry51Aa2.834_16 protein follows the same general steps as other *Bt* Cry proteins, including proteolytic activation, dimer dissociation, specific binding to brush border membranes and damage of the *Lygus* midgut [34].

It is important to note that these species were screened under a worst-case scenario of obligate and continuous consumption of the Cry51Aa2.834_16 protein at concentrations 1 to 2-fold of the highest expected environmental concentration (see Materials and Methods). Under this approach the realistic exposure routes and concentrations, as well as the feeding ecology of the organism were not considered. For example, to assess the potential risk from exposure of *O*. *insidiosus* to the Cry51Aa2.834_16 protein under relevant environmental conditions, further refined studies are required that determine a finite LC_50_ value and no effect concentration (NOEC), incorporate more realistic exposure concentrations (e.g. actual concentration of the Cry51Aa2.834_16 protein in the relevant tissue), and address the feeding ecology of the organism (i.e., secondary exposure through consumption of prey items).

When conducting the risk assessment for a transgenic crop, consideration must be given to assessing the potential risk of an insecticidal biotech crop/trait within the context of current risk posed by available practices for pest control [60]. Current methods for the control of *Lygus* spp. and other hemipteran and thysanopteran pests in cotton increasingly rely on the use of broad spectrum insecticides, which are known to have adverse effects on a variety of beneficial species including pollinators and natural enemies. In contrast with Cry proteins, the MoAs of these chemistries target biological systems common to or conserved across a broad spectrum of organisms (e.g. AChE inhibition from organophosphates and carbamates). Thus, adverse effects on a variety of pest and non-pest species have been reported [61, 62]. In contrast, the relatively narrow activity spectrum for Cry proteins has been shown to provide substantial health and environmental benefits, in locations where they are extensively used [63].

The results presented in this manuscript also have implications for resistance management of *Lygus* sp. as MON 88702 appears to be a less than high dose product targeting *Lygus*. Although the effective dose against *Lygus* is not high, there are key factors in *Lygus* ecology that will reduce the risk of resistance to Cry51Aa2.834_16. First, *Lygus* sp. have many alternative hosts that are utilized during the cotton-growing season [64] that will serve as refugia. Second, there is a great potential for movement between *Lygus* adults emerging from cotton and non-cotton hosts. Studies are ongoing to understand the dynamic ecology of *Lygus* with respect to resistance management to MON 88702.

The determination of Cry51Aa2.834_16 activity in these laboratory screening studies is not necessarily indicative of risk to tested species under field cultivation conditions for cotton expressing the Cry51Aa2.834_16 protein. Instead, the design of these screening studies was only intended to establish which species from a broad selection of NTOs are sensitive to the Cry51Aa2.834_16 protein, and can therefore inform the ERA for PIPs. The screening results presented for Cry51Aa2.834_16 demonstrate selective and limited activity only evident within three insect orders. Due to this specificity and the relatively low anticipated protein expression levels in cotton plants, the likelihood of adverse effects to non-target arthropods from a realistic environmental exposure to Cry51Aa2.834_16 protein expressed in MON 88702 is expected to be negligible.

## Supporting Information

S1 File Supporting information and data for the laboratory bioassays. Contains Figures A-D and Tables A-J.
(DOCX)
