# Supplementary material for: Characterization of the Activity Spectrum of MON 88702 and the Plant-Incorporated Protectant Cry51Aa2.834_16
Source: PLoS One. 2017 Jan 10;12(1):e0169409. doi: 10.1371/journal.pone.0169409 (PMC5224830; doi:10.1371/journal.pone.0169409)
Supplement: S1 File — Contains Figures A-D and Tables A-J. (DOCX) [file pone.0169409.s001.docx]

**Supporting Information**

**Fig A:** The mean six-day LC_50_ value from three bioassays for *L. hesperus* exposed to diet incorporated with Cry51Aa2.834_16 protein was determined to be 3.0 ± 0.4 µg Cry51Aa2.834_16 /ml diet. The concertation-response curves were generated using GraphPad PRISM ^®^, Prism 6 for Windows (GraphPad, 2013).


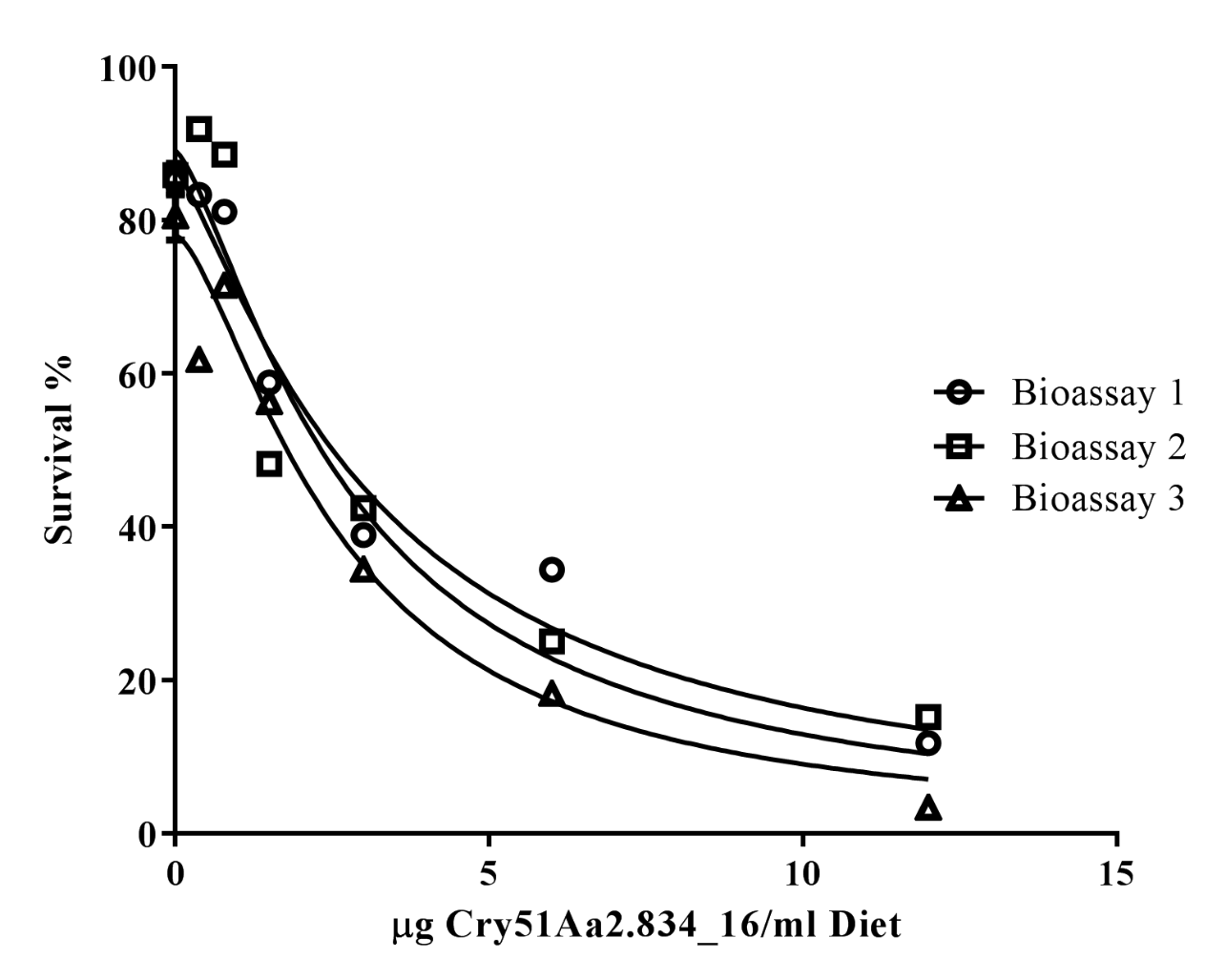


**Fig B:**  Cumulative development of *O. insidiosus* from continuous feeding studies with Cry51Aa2.834_16*.* The mean development time and its standard error on the 200 μg Cry51Aa2.834_16 /g diet treatment and 400 Cry51Aa2.834_16 μg/g diet treatment were not significant different from the buffer control, and were estimated to be 10.9 ± 0.2 d (p=0.149), and 10.9 ± 0.2 d (p=0.141), respectively.  Likewise, the mean development time on the buffer control treatment was estimated to be 10.5 ± 0.1 d. The development days to adult was statistically compared using an analysis of variance (ANOVA) (α = 0.05) conducted using SAS (SAS, 2012) running under Windows 7. The illustration of cumulative development was generated using GraphPad PRISM ^®^, Prism 6 for Windows (GraphPad, 2013)

**
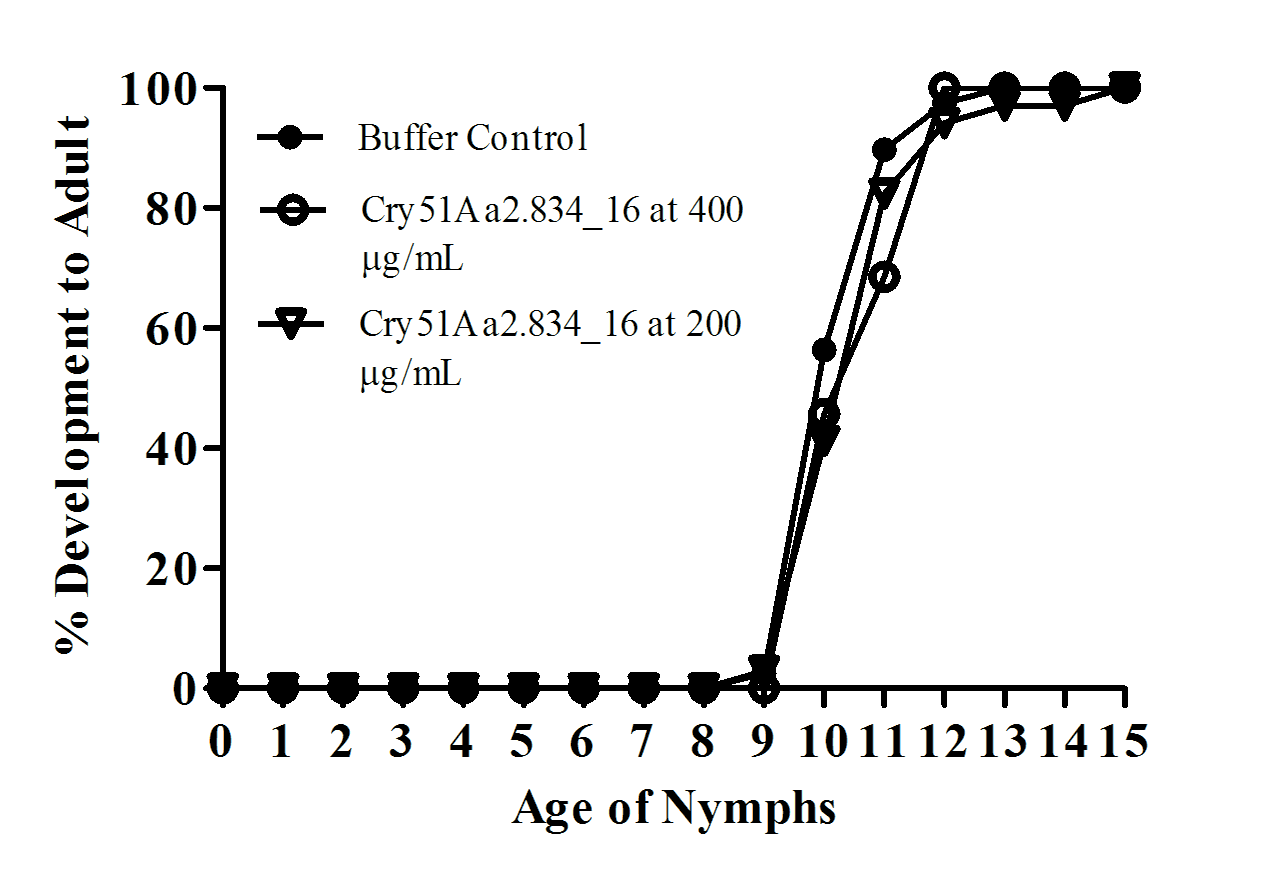
**

**Fig C:** Cumulative development of *C. maculata* from continuous feeding studies with Cry51Aa2.834_16. The mean development time and its standard error for C*. maculata* in the assay and buffer controls was 16.2 ± 1.3 d and 15.8 ± 1.1 d, respectively. Likewise, the mean development time in the Cry51Aa2.834_16 treatments was 15.9 ±1.1 d in the 200 µg /ml treatment and 16.1 ± 1 d in the 400 µg/ml treatment. The mean development time was not significantly different (p = 0.623) between the assay control, buffer control, and Cry51Aa2.834_16 treatments at 200 µg /ml and 400 µg/ml. The development days to adult were illustrated and statistically analyzed using ANOVA (α = 0.05), using GraphPad PRISM ^®^, Prism 6 for Windows (GraphPad, 2013).


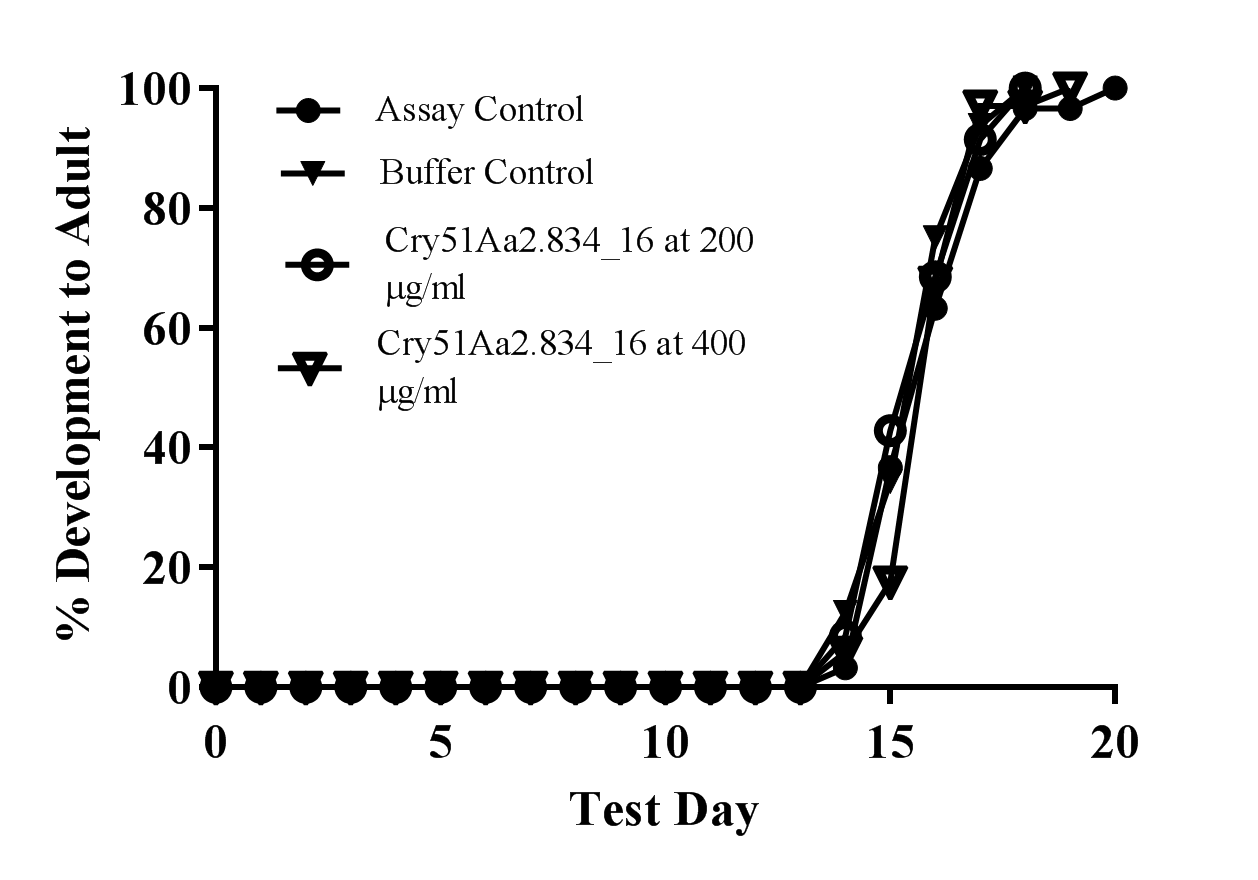


**Fig D**: Cumulative development of *A. mellifera* from continuous feeding studies with Cry51Aa2.834_16*.* The average development time and its standard error was estimated to be 14.3 ± 0.2 days in the Cry51Aa2.834_16 treatment, 14.3 ± 0.2 days in assay control treatment, and 14.7 ± 0.8 days in buffer control treatment. The mean development time on the Cry51Aa2.834_16 treatment was not significantly different from the assay control (p = 0.969) or the buffer control (p = 0.496). The development time of test honey bee larvae from dosing to adult emergence was calculated using SAS PROC MEANS program (SAS, 2012). SAS PROC MIXED was applied to fit model to conduct an analysis of variance (ANOVA) of the mean development time in each treatment. The pairwise comparisons between Cry51Aa2.834_16 and the assay control treatments and between Cry51Aa2.834_16 and the buffer control treatments were defined within the ANOVA and tested using t-test. The level of statistical significance for all tests was set at α = 0.05 and analysis was conducted using SAS (SAS, 2012) running under Windows 7. The illustration of cumulative development was generated using GraphPad PRISM ^®^, Prism 6 for Windows (GraphPad, 2013)

**
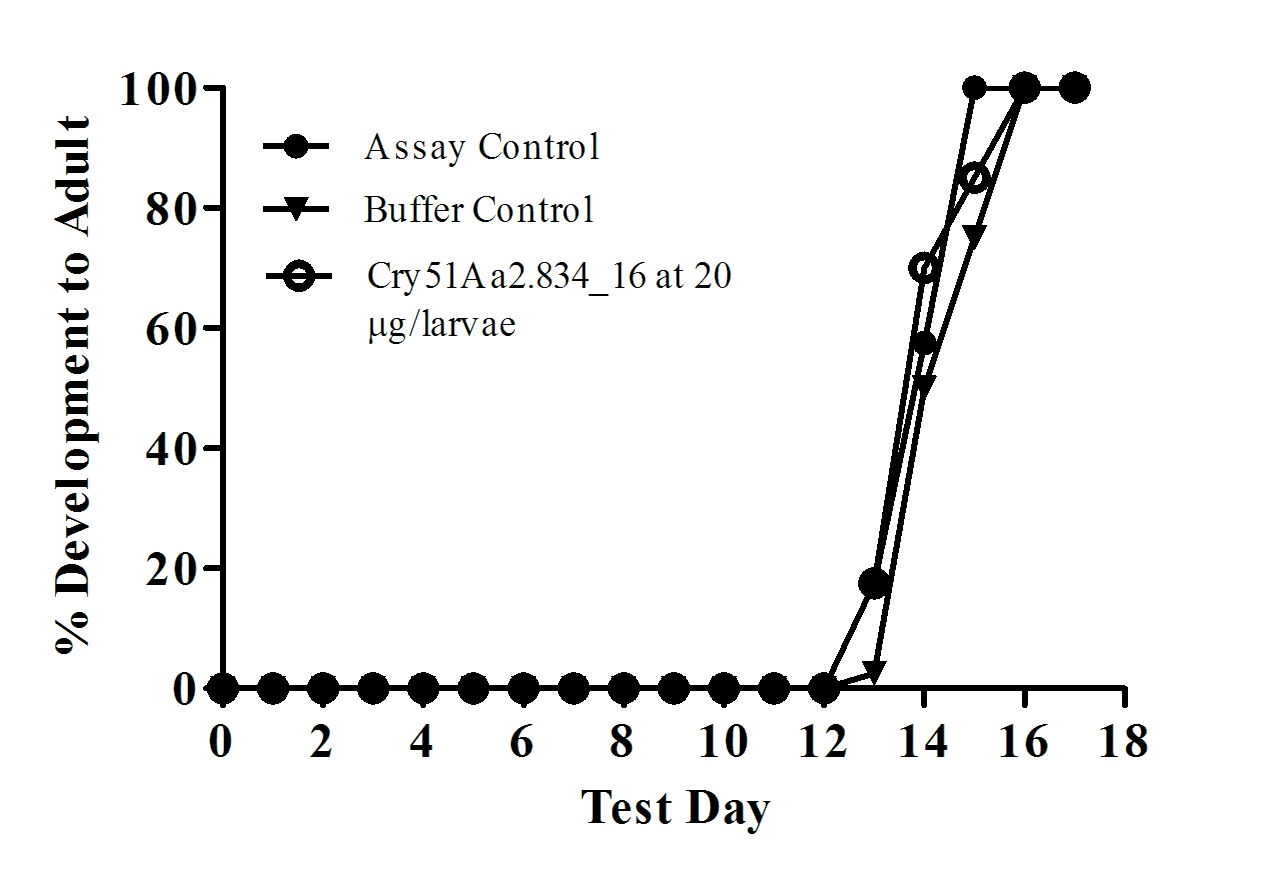
**

**Table A:** LC_50_ estimates for the Cry51Aa2.834_16 protein from three bioassays for *L. hesperus*. PROBIT analysis with SAS (SAS, 2012), a statistical program running under Windows 7, estimated the dose level required for 50% mortality (LC_50_).

| Bioassay Replicate | **LC_50 (_μg/mL diet)** | **95% Confidence Intervals**  **(μg/ml diet)** |
| --- | --- | --- |
| 1 | 3.3 | 2.3 - 4.6 |
| 2 | 3.2 | 1.4 - 6.6 |
| 3 | 2.6 | 1.2 - 3.7 |
| Mean LC_50_: 3.0 μg /ml diet  SD: 0.4 μg /ml diet | | |

**Table B:** Survival of *O. insidiosus* from continuous feeding studies with Cry51Aa2.834_16. The survival results were analyzed for significant differences using Fisher’s exact test (α = 0.05) conducted using SAS (SAS, 2012) running under Windows 7.

| **Treatment** | **Percent Survival** | **Mean Time to Adult (d)** |
| --- | --- | --- |
| Buffer Control | 98 | 10.5 ± 0.1 |
| 200 μg Cry51Aa2.834_16/g diet | 67 | 10.9 ± 0.2 |
| 400 μg Cry51Aa2.834_16/g diet | 67^1^ | 10.9 ± 0.2 |
| 100 μg KH_2_AsO_4_ /ml | 0 | N/A |

^1^Significantly different from buffer control at α = 0.05 (p = 0.001)

**Table C:** Survival of *L. decemlineata,* *D. u. howardi*, and *D. v. virgifera* from continuous feeding studies with Cry51Aa2.834_16. The survival results were analyzed for significant differences using Fisher’s exact test (α = 0.05). Statistical Analysis was performed using GraphPad PRISM ^®^, Prism 6 for Windows (GraphPad, 2013).

| **Test Organism** | **Treatment** | **Percent Survival**  **at Day 7** | **Percent Survival**  **at Day 12** |
| --- | --- | --- | --- |
| *L. decemlineata* | Assay Control | 92 | - |
|  | Buffer Control | 92 | - |
|  | 200 µg Cry51Aa2.834_16/ml | 50 | - |
|  |  |  |  |
| *D. u.howardi* | Buffer Control^1^ | - | 85 |
|  | 200 µg Cry51Aa2.834_16/ml | - | 55^3^ |
|  |  |  |  |
| *D. v.virgifera* | Buffer Control^1^ | 97 | 74 |
|  | 1000 µg Cry51Aa2.834_16/ml | 100 | 78 |

^1^The buffer control treatment represents mean survival of three replicates.

^2^ Significantly different from combined control at α = 0.05 (p < 0.001)

^3^ Significantly different from buffer control at α = 0.05 (p = 0.001)

**Table D:** Survival and development from feeding studies with Cry51Aa2.834_16 for *C. maculata* . The survival/mortality results, and development to the adult stage, were analyzed for significant differences (α = 0.05) with Fisher’s exact test using SAS ([SAS, 2012](#_ENREF_11)) running under Windows 7. The development days to adult and adult mass endpoints were statistically analyzed with ANOVA (α = 0.05), using GraphPad PRISM ^®^, Prism 6 for Windows (GraphPad, 2013)..

| **Treatment** | **Percent Survival** | **Percent Adult Emergence** | **Larval Development Time (d)**  **Mean ± SE** | **Adult Weight (mg)**  **Mean ± SE** |
| --- | --- | --- | --- | --- |
| Assay Control | 78 | 78. | 16.2 ± 1.3 | 9.2 ± 1.6 |
| Buffer Control | 82 | 82 | 15.8 ± 1.1 | 9.7 ± 1.4 |
| 200 µg Cry51Aa2.834_16/ml | 88 | 88 | 15.9 ± 1.1 | 9.7 ± 1.0 |
| 400 µg Cry51Aa2.834_16/ml | 93 | 85 | 16.1 ± 1.0 | 9.6 ± 1.6 |
| KH_2_AsO_4_ | 35 | 0 | -- | -- |

**Table E:** Survival from feeding studies with Cry51Aa2.834_16 for *E. varivestis.* The survival results were analyzed for significant differences (α = 0.05) with Fisher’s exact test using SAS ([SAS, 2012](#_ENREF_11)) running under Windows 7.

| **Treatment** | **Percent Survival**  **at Day 7** | **Percent Survival**  **at Day 14** |
| --- | --- | --- |
| Assay Control | 100 | 90 |
| Buffer Control | 98 | 93 |
| 200 µg Cry51Aa2.834_16/ml | 100 | 98 |
| 400 µg Cry51Aa2.834_16/ml | 100 | 95 |
| 14 µg KH_2_AsO_4_/ml | 78 | 40 |
| 28 µg KH_2_AsO_4_/ml | 73 | 3 |

**Table F:** Survival from feeding studies with Cry51Aa2.834_16 for *O. nubilalis*, *H. zea*, *S. frugiperda*, and *P. xylostella*. The survival results were analyzed for significant differences using Fisher’s exact test (α = 0.05) using GraphPad PRISM ^®^, Prism 6 for Windows (GraphPad, 2013).

| **Lepidoptera Species** | **Treatment^1^** | **Percent Survival**  **at Day 7** |
| --- | --- | --- |
| *O. nubilalis* | Assay Control | 100 |
|  | Buffer Control | 100 |
|  | 400 µg Cry51Aa2.834_16/ml | 97 |
|  |  |  |
| *H. zea* | Assay Control | 94 |
|  | Buffer Control | 100 |
|  | 400 µg Cry51Aa2.834_16/ml | 97 |
|  |  |  |
| *S. frugiperda* | Assay Control | 100 |
|  | Buffer Control | 94 |
|  | 400 µg Cry51Aa2.834_16/ml | 100 |
|  |  |  |
| *P. xylostella* | Assay Control | 100 |
|  | Buffer Control | 100 |
|  | 400 µg Cry51Aa2.834_16/ml | 100 |

^1^The Cry51Aa2.834_16 treatment includes the combined results of two replicates.

**Table G**: Survival data from feeding studies with Cry51Aa2.834_16 for *P. foveolatus* . The survival results were analyzed for significant differences using Fisher’s exact test (α = 0.05) using SAS (SAS, 2012) running under Windows 7.

| **Treatment** | **Percent Survival** |
| --- | --- |
| Assay control | 100 |
| Buffer control | 97 |
| 200 μg Cry51Aa2.834_16/ml | 100 |
| 400 μg Cry51Aa2.834_16/ml | 94 |
| 200 μg KH_2_AsO_4_/ml | 0 |

**Table H:** Survival and emergence times from feeding studies with Cry51Aa2.834_16 for *A. mellifera.*  A statistical analysis was not performed since there was 100% survival in the Cry51Aa2.834_16 treatment, assay control, and buffer control. However, development time of test honey bee larvae from dosing to adult emergence was calculated using SAS PROC MEANS program [41]. SAS PROC MIXED was applied to fit model to conduct an analysis of variance (ANOVA) of the mean development time in each treatment. The pairwise comparisons between Cry51Aa2.834_16 and the assay control treatments and between Cry51Aa2.834_16 and the buffer control treatments were defined within the ANOVA and tested using t-test. The level of statistical significance for all tests was set at α = 0.05 using SAS (SAS, 2012) running under Windows 7.

| **Treatment** | **Percent Survival** | **Mean Time to Adult Emergence (d)** |
| --- | --- | --- |
| Assay Control | 100 | 14.3 ± 0.2 |
| Buffer Control | 100 | 14.7 ± 0.8 |
| 20 μg Cry51Aa2.834_16/larvae^1^ | 100 | 14.3 ± 0.2 |
| 20 μg KH_2_AsO_4_ /larvae^2^ | 0 | NA |

^1^ μg Cry51Aa2.834_16⁄larva= (2000μg Cry51Aa2.834_16⁄ml diet solution)×(10 μl⁄ larval cell)=20 μg Cry51Aa2.834_16⁄larva

^2^ μg KH_2_AsO_4_⁄larva=(2000 μg KH_2_AsO_4_⁄ml diet solution)×(10 μl⁄ larval cell)=20 μg KH_2_AsO_4_⁄larva

**Table I:** Survival and reproduction from feeding studies with Cry51Aa2.834_16 for *F. candida.* The numbers of surviving adults were used to calculate the percentage mortality of the *F. candida* originally introduced in each treatment. The 28-day mortality data for the test-substance treatment was compared to that for both the buffer control and the assay control using Fisher’s exact test (α = 0.05) while the number of progeny were statistically compared using an analysis of variance (ANOVA) with a square root transformation (α = 0.05). Both analyses were conducted using SAS (SAS, 2012) running under Windows 7.

| **Treatment** | **Percent Mortality  at 28 Days** | **Percent Survival at 28 Days** | **Number of Progeny** |
| --- | --- | --- | --- |
|  |  |  | **Mean ± SE** |
| Assay Control | 0 | 100 | 170 ± 2 |
| Buffer Control | 0 | 100 | 161 ± 2 |
| Blank (starvation) control | 0^+^ | 100 | 2 ± 0 |
| 400 µg Cry51Aa2.834_16/g^1^ | 3 | 97 | 159 ± 7 |
| Toxic reference diet | 83^+^ | 17 | 4 ± 0 |

^1^The Cry51Aa2.834_16 treatment includes the combined results of two replicates.

+ Surviving springtails still very small, when compared to the buffer and assay control treatments.

**Table J:** Survival and biomass data from feeding studies with Cry51Aa2.834_16 for *E. andrei*. The percentage mortality in the test-item treatment, buffer control and water control were compared with one another using Fisher’s exact test (α = 0.05) while the percentage change in weight of the worms for the two controls and test-item treatment over the 14-day bioassay was were statistically compared using an analysis of variance (ANOVA) (α = 0.05). Both analyses were conducted using SAS (SAS, 2012) running under Windows 7.

| **Treatment** | **Percent Mortality Adult Worms  at 14 Days** | **Percent Survival Adult Worms at 14 Days** | **Percent Change in Adult Worm Fresh Weight ^1^**  **Mean ± SE** |
| --- | --- | --- | --- |
| Assay Control | 0 | 100 | -9.4 ± 0.4 |
| Buffer Control | 0 | 100 | -6.7 ± 0.7 |
| 400 µg Cry51Aa2.834_16/g soil dw | 0 | 100 | -6.2 ± 0.8 |

^1^The mean (and SE) for change in adult worm weight in each arena between day 0 and day 14. A negative value indicates a decrease in fresh weight.

**References**

GraphPad PRISM ^®^, Prism 6 for Windows Version 6.03, © 1992-2013 GraphPad Software, Inc. La Jolla, California. 2013.

SAS. Software Release 9.4 (TS1M1). Copyright 2002-2012 by SAS Institute, Inc., Cary, North Carolina. 2012.
